# HOME MODIFICATION ON FAVORITE ACTIVITY AND SELF-EFFICACY AMONG OLDER ADULTS WITH DEMENTIA

**DOI:** 10.1093/geroni/igad104.3392

**Published:** 2023-12-21

**Authors:** Soobin Park, Eunsun Kwon, Sojung Park, Oejin Shin, Sehyun Baek

**Affiliations:** Washington University in Saint Louis, St. Louis, Missouri, United States; Fairleigh Dickinson University, Teaneck, New Jersey, United States; Washington University in Saint Louis, St. Louis, Missouri, United States; Illinois State University, Normal, Illinois, United States; Washington University in Saint Louis, St. Louis, Missouri, United States

## Abstract

Globally, most older people with dementia live at home and want to remain at home as long as possible with a sense of agency, and engagement in preferred everyday activities. To remain at home, a safe and supportive home environment is crucial as dementia progresses. Yet research on environmental modification among people with dementia is focused on neighborhood or urban built environment, and less on home modification. Guided by the ecological model of aging, this study aimed to explore the impact of home modification on both affective (e.g., self-efficacy) and adaptive behavioral (e.g., favorite activities) responses as a result of person-environmental fit among people living with dementia. Using two waves (2018-2019) data from the National Health and Aging Trends Study (NHATS), we examined the extent to which home modification is associated with self-efficacy and engagement in favorite activities. Multi-group structural equation modeling was conducted on 273 older adults living with dementia in the communities, comparing 145 with functional limitations and 129 without. Although home modification did not affect one’s self-efficacy in any group, a positive impact of home modification on engaging favorite activities, especially those that require more physical effort (e.g., active leisure), was significant among older people with dementia with functional difficulties (p< 0.01). Our finding points to the importance of home modification for the most vulnerable subgroup of older adults with dementia, not only ensuring physical safety at home but their continued engagement in the activities meaningful for them, thereby helping them age in place.

